# Importance of ozone precursors information in modelling urban surface ozone variability using machine learning algorithm

**DOI:** 10.1038/s41598-022-09619-6

**Published:** 2022-04-05

**Authors:** Vigneshkumar Balamurugan, Vinothkumar Balamurugan, Jia Chen

**Affiliations:** 1 Environmental Sensing and Modeling, Technical University of Munich (TUM), 80333 Munich, Germany; 2 Mechanical Engineering, St. Joseph’s Institute of Technology, Chennai, 600119 India

**Keywords:** Environmental sciences, Atmospheric chemistry

## Abstract

Surface ozone (O$_3$) is primarily formed through complex photo-chemical reactions in the atmosphere, which are non-linearly dependent on precursors. Even though, there have been many recent studies exploring the potential of machine learning (ML) in modeling surface ozone, the inclusion of limited available ozone precursors information has received little attention. The ML algorithm with in-situ NO information and meteorology explains 87% (R$^{2}$ = 0.87) of the ozone variability over Munich, a German metropolitan area, which is 15% higher than a ML algorithm that considers only meteorology. The ML algorithm trained for the urban measurement station in Munich can also explain the ozone variability of the other three stations in the same city, with R$^{2}$ = 0.88, 0.91, 0.63. While the same model robustly explains the ozone variability of two other German cities’ (Berlin and Hamburg) measurement stations, with R$^{2}$ ranges from 0.72 to 0.84, giving confidence to use the ML algorithm trained for one location to other locations with sparse ozone measurements. The inclusion of satellite O$_3$ precursors information has little effect on the ML model’s performance.

## Introduction

In today’s world, air quality is a major environmental threat to human health; additionally, some key air pollutants, either directly or indirectly, contribute to climate change (https://www.who.int/). Despite the fact that anthropogenic emissions of key air pollutants have decreased significantly as a result of stringent emission control measures implemented over the last two decades, air quality in many parts of Europe remains poor^1^. Particularly, secondary air pollutants (ozone, secondary particulate matter) formed by complex atmospheric photo-chemical reactions did not show the same trend of decreasing as primary air pollutants, which are emitted directly from primary sources^2^. Ozone (O$_3$) has a negative impact on both human health and the ecosystem^3,4^, and also a potent greenhouse gas. The primary source of ozone in the troposphere is photolysis of nitrogen dioxide (NO$_2$). Volatile organic compounds (VOCs) play a larger role in ozone production through producing hydrogen oxide radicals (HO$_X$ = OH + HO$_2$ + RO$_2$) (catalytic cycle), which drive the conversion of NO to NO$_2$ (NO$_X$ = NO + NO$_2$)^5,6^. Because of the termination reactions that occur during the catalytic cycle, ozone production is not always directly proportional to the precursor’s emission or concentration (NO$_X$ and VOC)^7,8^. As a result, ozone production is widely classified into three regimes: NO$_X$ limited (low NO$_X$ and high VOC), NO$_X$ saturated (high NO$_X$ and low VOC), and transitional^9,10^. Ozone production can be controlled by lowering NO$_X$ in a NO$_X$ limited regime, whereas lowering NO$_X$ can increase ozone production in a NO$_X$ saturated regime. The major source of NO$_X$ in the urban environment is traffic, whereas VOC from traffic is minor, but biogenic VOC emissions are significant^1,11^. Meanwhile, VOC emissions from volatile chemical products such as cleaning agents and personal care products are becoming more significant^12^. Recent ozone enhancements in urban areas during the COVID-19 lockdown period demonstrate the NO$_X$ saturated regime’s ozone production chemistry^13,14^. Chemical transport models (CTM) are widely used to study the ozone variability^15–19^. However, CTMs have a large bias in resolving complex topography and chemistry mechanisms due to coarser resolution^20,21^, for example, urban areas are typically in a NO$_X$ saturated regime, whereas rural areas are being in a NO$_X$ limited regime. In addition, the bias in CTM is exacerbated when emission inventories are uncertain^22^. CTM, on the other hand, necessitate massive computational resource.

Machine learning (ML) is gaining traction as an alternative modeling tool to complement CTM in Earth system science fields^23–29^. Because photo-chemical processes have a significant impact on ozone, ML algorithms are trained using a wide range of meteorological variables, many of which drive photo-chemical processes^30–36^. The variability of surface ozone is well explained by the ML algorithm with meteorological information alone^37–39^. Temperature is identified as a key factor in explaining ozone variability in the ML model^40^. Temperature is also a driver of biogenic VOC emissions (a precursor to O$_3$) in addition to being a driver of photo-chemical processes^7,8^. In the NO$_X$ saturated regime, ozone production is directly proportional to VOC emission (and thus to temperature), but in the NO$_X$ limited regime, ozone dependency on VOC shifts to NO$_X$^41^. Given that many urban areas are currently in a NO$_X$ saturated regime, it is reasonable to expect that ML algorithm trained solely on meteorology will be able to explain ozone variability. After transitioning to a NO$_X$ limited regime, the ML algorithm trained solely on meteorology may fail to reproduce the surface ozone variability. Previous studies have also shown that the ozone response to temperature has been decreasing in recent years, as urban regions are transitioning to NO$_X$ limited regime^42,43^. However, only a few studies have focused on the inclusion of precursor information into the ML model^33,34,36^.

In-situ VOC and O$_3$ measurements are too scarce when compared to NO$_X$ measurements, and all are even scarcer in rural areas. Satellite data are becoming an indispensable tool for analyzing urban and rural air quality due to their increasing spatial resolution and spatial coverage, but they are column retrievals. Since stratospheric ozone is highly variable, total column ozone retrievals from satellites are unsuitable for studying surface ozone. Satellites, on the other hand, retrieve the ozone precursors (NO$_2$ and HCHO (formaldehyde)), which can be used to study the surface ozone chemistry^44–46^. Because HCHO is an intermediate gas-product of VOC oxidation, it can be used as a proxy for VOC emissions. As CTMs resolve the physical-chemical processes, whereas ML algorithms do not, a hybrid modelling approach that incorporates the CTM prediction as a predictor variable into the ML model may improve the performance^47^. To this end, the objectives of this study are formulated as follows: 1) investigate the importance of limited available (in-situ and satellite) ozone precursor information and coarse CTM ozone simulations in modeling urban surface ozone variability using ML algorithm; and 2) investigate the potential of ML model’s transfer-ability; how well the ML algorithm trained for one location explains ozone variability in other locations. The ultimate goal of these two objectives is to provide us confidence in modeling the surface ozone variability of locations with sparse or no ozone measurements and filling the data gap.

## Study region, datasets and model

This study focuses on Munich, a southern German metropolitan area where air pollutants are currently measured at five different locations. Given the long-term availability of all pollutants data, we chose an urban measurement station (Lothstrasse) to train and test the ML model, which continuously measured O$_3$, NO$_2$, NO, and CO from 2001 to 2017. In our study, we also used data (2003 to 2017) from other three stations in Munich (Johanneskirchen-suburban, Allach-suburban, and Stachus-urban) to assess the transfer-ability of the ML model. We also tested the ML model’s transfer-ability using data (2015 to 2019) from measurement stations in other German cities, including Berlin (Neukollen-urban, Wedding-urban, and Buch-suburban) and Hamburg (Bramfeld-suburban, Neugraben-suburban, and Sternschanze-urban). The geographical locations of three German metropolitan areas (Munich, Berlin and Hamburg) and its monitoring stations considered in this study are shown in Fig. S1.

Meteorological variables (temperature, boundary layer height, relative humidity, wind speed and wind direction) are obtained from the ERA 5 reanalysis dataset, with spatial and temporal resolutions of 0.25° and 1 h, respectively^48^. Surface ozone simulations of CAMS (Copernicus Atmosphere Monitoring Service) global reanalysis dataset (EAC4) are also obtained from CAMS data store, which has a spatial resolution of 0.75° and a temporal resolution of 3 h.

The tropospheric column NO$_2$ and HCHO data from the NASA Aura satellite’s OMI (ozone monitoring instrument) are also used^49^. OMI data has a spatial resolution of 13 * 24 km and a daily temporal resolution. The OMI local overpass occurs between 1 p.m. and 2 p.m. OMI data are available beginning in October of 2004. We filtered the OMI data before using it to include only data with no processing errors, less than 10% snow or ice cover, a solar zenith angle of less than 80° for NO$_2$ (70° for HCHO), and a cloud radiance fraction of less than 0.5. At the end, we only had 689 days of OMI data out of 4809 days (October, 2004 to December, 2017) for “Lothstrasse” station.

The Extreme Gradient Boosting (XGBoost) algorithm, a supervised learning-gradient boosting tree-based ML algorithm^50^, is used in this study to model surface ozone concentrations. Since our objective is to investigate the importance of precursor information in surface ozone modeling using ML, the ML algorithm we choose should be more interpretable. A tree-based ML algorithm, such as XGBoost, is more interpretable than neural networks, which are typically black box systems, and also achieves higher interpretability than simple linear regression algorithms (high-bias algorithm)^51^. We train the XGBoost ML algorithm with different predictor categories or combinations of predictor categories (Table 1), and then compare its performance in terms of correlation (R$^{2}$) and root mean square error (RMSE). The predictor categories are broadly classified into meteorology (temperature, relative humidity, boundary layer height, wind speed and wind direction), in-situ ozone precursors (NO, NO$_2$ and CO), satellite ozone precursors (column NO$_2$ and HCHO) and CTM simulations (CAMS model surface O$_3$). Additionally, we consider two more predictors (day of the week and season), which we include in the meteorology category. The hyper-parameters of the XGBoost algorithm (such as the number of gradient boosted trees, learning rate, and maximum depth of a tree, etc.) are tested using grid search function (https://scikit-learn.org/stable/modules/generated/sklearn.model_selection.GridSearchCV.html) and, we find that XGBoost algorithm is not sensitive to hyper-parameters in this study. Therefore, the hyper-parameters were set to their default values (https://xgboost.readthedocs.io/en/latest/parameter.html). We also discuss the predictor variable (feature) importance in the ML model using the results derived from sklearn python library’s “feature_importance” function, which calculates feature importance by taking the average gain across all splits (https://scikit-learn.org/stable/auto_examples/ensemble/plot_gradient_boosting_regression.html). For this study, we focus on the afternoon (1 p.m. to 2 p.m.) when ozone levels are at their highest (diurnal maximum), matching with the OMI satellite overpass time. We also performed a similar analysis with the Random Forest (RF) ML algorithm.

**Table 1:** Different ML simulation type and associated training data (marked as X).

ML simulation name	Predictor variables					Result		
	Meteorology		In-situ ozone precursors measurement	Satellite ozone precursors retrieval	CTMs simulation			
	T, RH, BLH, WS, WD	DW, S	Surface NO, NO$_2$, CO	Tropospheric column NO$_2$, HCHO	CAMS surface O$_3$ simulations	R$^{2}$	RMSE	Mean R$^{2}$ of K(10)-fold CV
ML_met (1)	X					0.74	18.1	0.72
ML_met_ds (2)	X	X				0.76	17.5	0.74
ML_cams (3)					X	0.64	21.6	0.63
ML_insitu (4)			X			0.47	26.1	0.49
ML_met_ds_insitu (5)	X	X	X			0.80	15.8	0.81
ML_met_ds_insitu_cams (6)	X	X	X		X	0.83	14.9	0.84
ML_satellite (7)				X		− 0.35	41.7	-0.31
ML_met_ds_satellite (8)	X	X		X		0.77	17.1	0.74
ML_met_ds_satellite_cams (9)	X	X		X	X	0.81	15.6	0.80

T-Temperature, RH-Relative Humidity, BLH-Boundary Layer Height, WS-Wind Speed, WD-Wind Direction, DW-Day of Week, S-Season, NO-Nitric oxide, NO$_2$-Nitrogen Dioxide, CO-Carbon Monoxide, O$_3$-Ozone and HCHO-Formaldehyde. The index of different ML simulation types is given in brackets in the first column, to which we refer in Fig. 2. The performance of each ML simulation with fewer days case (689 days) at lothstrasee station is shown in the last three columns.

## Results

### Performance of ML model in predicting the urban surface ozone

**Figure 1:**
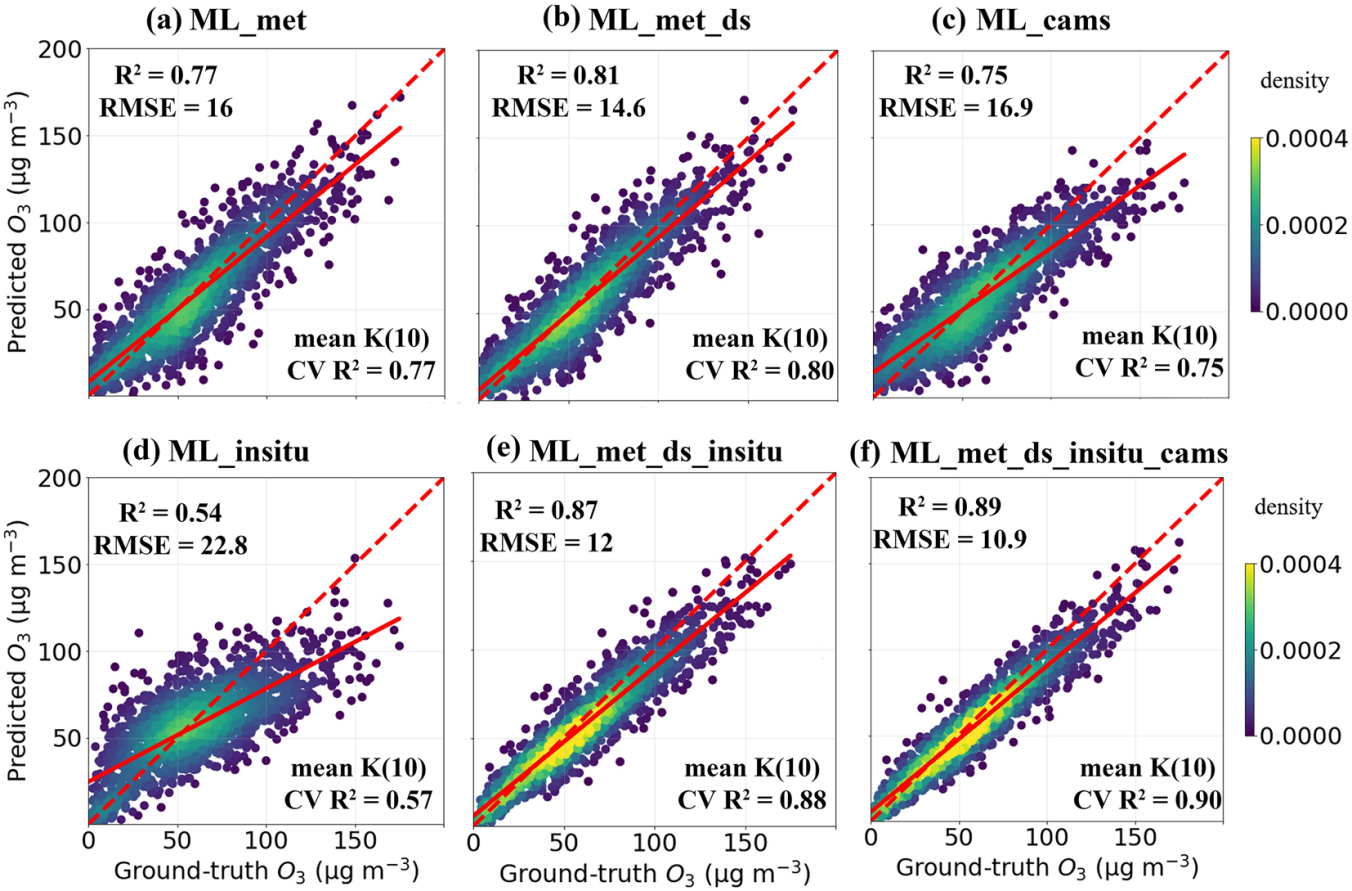
Density scatter plots of predicted ozone by different ML simulation type vs ground-truth ozone at Lothstrasse station at Munich. In a total of 5375 days (between 2001 to 2017), first 3800 days used for training and remaining 1575 days used for testing. Mean R$^{2}$ of k(10)-fold cross validation is also given at bottom of figure panels at each case. Red solid line represents the linear fit and red dotted line represents 1:1 line.

For the “Lothstrasse” station in Munich, all in-situ measurements, meteorological variables and CAMS data are available for 5375 days from 2001 to 2017. We divided the 5375 days of measurements into two parts: first 3800 days (70%) for training, and remaining 1575 days (30%) for testing the ML predictions. The k-fold cross validation (CV) is used to evaluate the performance of the ML model for different dataset combinations for training and testing. Here we choose k as 10, i.e., 5375 days of data split into 10 parts. To avoid spurious correlation between training and test datasets, we adopted a block sampling approach^52^. The first nine parts are used to train the ML algorithm, and the final one is used to test the ML model; this process is repeated ten times for the remaining combinations. The mean of R$^{2}$ derived from the k(10)-fold cross validation is then computed. The ML algorithm that was trained solely on meteorology (“ML_met”) explains 77 percent of the variance (R$^{2}$ = 0.77) in measured O$_3$, with RMSE of 16 $\upmu$g m$^{-3}$ (Fig. 1a). The mean R$^{2}$ of k(10)-fold CV is 0.77. Wind speed and wind direction have a low importance in the fitted model when compared to other meteorological variables (relative humidity, boundary layer height, and temperature) (Fig. S2). In addition, including the day of the week and season in the training dataset (“ML_met_ds”) improves the ML model’s performance (R$^{2}$ = 0.81, RMSE = 14.6 $\upmu$g m$^{-3}$ and mean R$^{2}$ of k(10)-fold CV = 0.80) (Fig. 1b). This performance improvement could be attributed to the pronounced seasonal cycle of ozone and weekday-weekend differences. The ozone reaches its maximum in summer and minimum in winter, and due to being in a NO$_X$ saturated regime, weekend ozone levels are higher than the weekdays^13^. The ML algorithm trained solely with CAMS (“ML_cams”) or in-situ precursors (“ML_insitu”) show poor performance in all terms when compared to ML algorithm trained with the meteorology category alone (“ML_met_ds”) (Fig. 1c,d).

The ML algorithm trained with meteorology and in-situ precursors category (“ML_met_ds_insitu”) performs better than “ML_met_ds”, with R$^{2}$ and RMSE are about 0.87 and 12 $\upmu$g m$^{-3}$, respectively (Fig. 1e). The scatter of predicted O$_3$ by “ML_met_ds” is largely reduced in “ML_met_ds_insitu”, resulting in a lower RMSE. The mean R$^{2}$ of k(10)-fold CV is 0.88, which is a 15% increase over “ML_met”. The important feature in “ML_met_ds_insitu” is derived to be in-situ NO measurements, followed by boundary layer height, temperature, and relative humidity. The improvement in performance from “ML_met_ds_insitu” is thus due to the inclusion of NO measurements in the model. The addition of CAMS O$_3$ simulations with meteorology and in-situ precursors (“ML_met_ds_insitu_cams”) further improves the model performance (R$^{2}$ = 0.89, RMSE = 10.9 $\upmu$g m$^{-3}$ and mean R$^{2}$ of k(10)-fold CV = 0.9), which is slightly higher than that of “ML_met_ds_insitu” (Fig. 1f), with CAMS O$_3$ simulations being the most important feature (Fig. S2). The feature importance calculated using the permutation approach (https://christophm.github.io/interpretable-ml-book/feature-importance.html) and SHAP values (https://christophm.github.io/interpretable-ml-book/shap.html) agree with the feature importance calculated using each feature’s gain. For example, Fig. S3 shows the feature importance calculated based on permutation and SHAP values for the case ”ML_met_ds_insitu”. We also performed a similar analysis using Random Forest ML algorithm with a split of 5375 dataset into 70%/30% (training/testing) (Table S1). When compared to “ML_met_ds” in RF model simulations, the performance of “ML_met_ds_insitu” is improved (in all terms). This supports our earlier findings that including in-situ precursor information is not redundant when modeling surface ozone with ML model.

**Figure 2:**
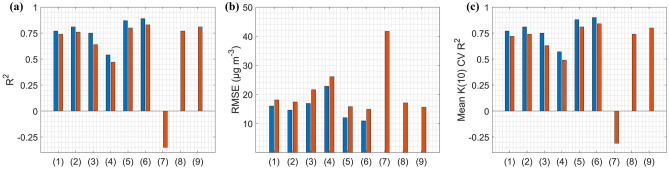
Performance comparison of different ML simulation types with 5375 days (blue) and 689 days (red) for training and testing. X axis indexes refer to the index of different ML simulation type (Table 1).

For 689 days between 2001 and 2017, all in-situ and satellite ozone precursors information, meteorological variables and CAMS data are available. Similarly, we use the first 70% of data (480 days) for training and remaining 30% (209 days) for testing the model. Also, we performed the k(10)-fold CV for 689 days of dataset. The performance of the ML algorithm trained with meteorology and satellite precursors (“ML_met_ds_satellite”) is, however, equal to the performance of the ML algorithm trained with meteorology alone (Fig. 2a–c). This implies that including satellite ozone precursor data had less effect on model performance. In terms of mean R$^{2}$ of k(10)-fold CV, the ML algorithm with meteorology, satellite precursors, and the CAMS category provides slightly better results. However, it is poor than that of the ML algorithm trained with meteorology, in-situ precursors, and the CAMS category. The performance difference between ML model with a high (5375) and low (698) number of days is marginal. In all cases, the performance of the ML model with fewer days (698 days) is slightly worse than the performance of the ML model with 5375 days for training and testing (Fig. 2a–c). To see how the availability of training dataset affects performance, we train and test the “ML_met_ds_insitu” for varying percentages of data for the 5375 days case (Fig. S4). The difference between different dataset combinations for training and testing is also marginal; the 80%/20% (training/testing) dataset performs slightly better than the 20%/80% dataset (lower RMSE by 1.5 $\upmu$g m$^{-3}$ and higher R$^{2}$ by 0.03). However, in this case, 20% of data equates to nearly three years of data, which may be sufficient to capture all ozone variability by ML model.

We investigated the sensitivity of each predictor variable in the ML model. This is done by excluding the particular predictor variable from the “ML_met_ds_insitu” (Table S2). Temperature is the important feature fitted in model. When temperature is excluded from “ML_met_ds_insitu”, the RMSE increases by 1.9 $\upmu$g m$^{-3}$ and the R$^{2}$ decreases by 0.04 compared to all variables included in “ML_met_ds_insitu”. Furthermore, at each case, when variable such as season, relative humidity, wind direction, boundary layer height, and in-situ NO is excluded, RMSE increases and R$^{2}$ decreases. There are no changes in RMSE and R$^{2}$ when the day of the week or wind speed is removed. When in-situ NO$_2$ or CO is removed, the RMSE decreases in comparison to “ML_met_ds_insitu”, indicating that the model is over-fitted when these variables are included. Therefore, we train the ML algorithm only with season, relative humidity, temperature, wind direction, boundary layer height and in-situ NO variables (“ML_s_rh_t_wd_blh_no”), which show slightly better performance in-terms of RMSE decreases by 0.4 $\upmu$g m$^{-3}$ compared to “ML_met_ds_insitu”. Figure S5 depicts a time series plot of ground-truth vs modeled surface ozone concentrations, demonstrating the ML model’s superior performance in modeling complex ozone variability ranging from daily to seasonal variation.

### ML model’s transfer-ability

**Figure 3:**
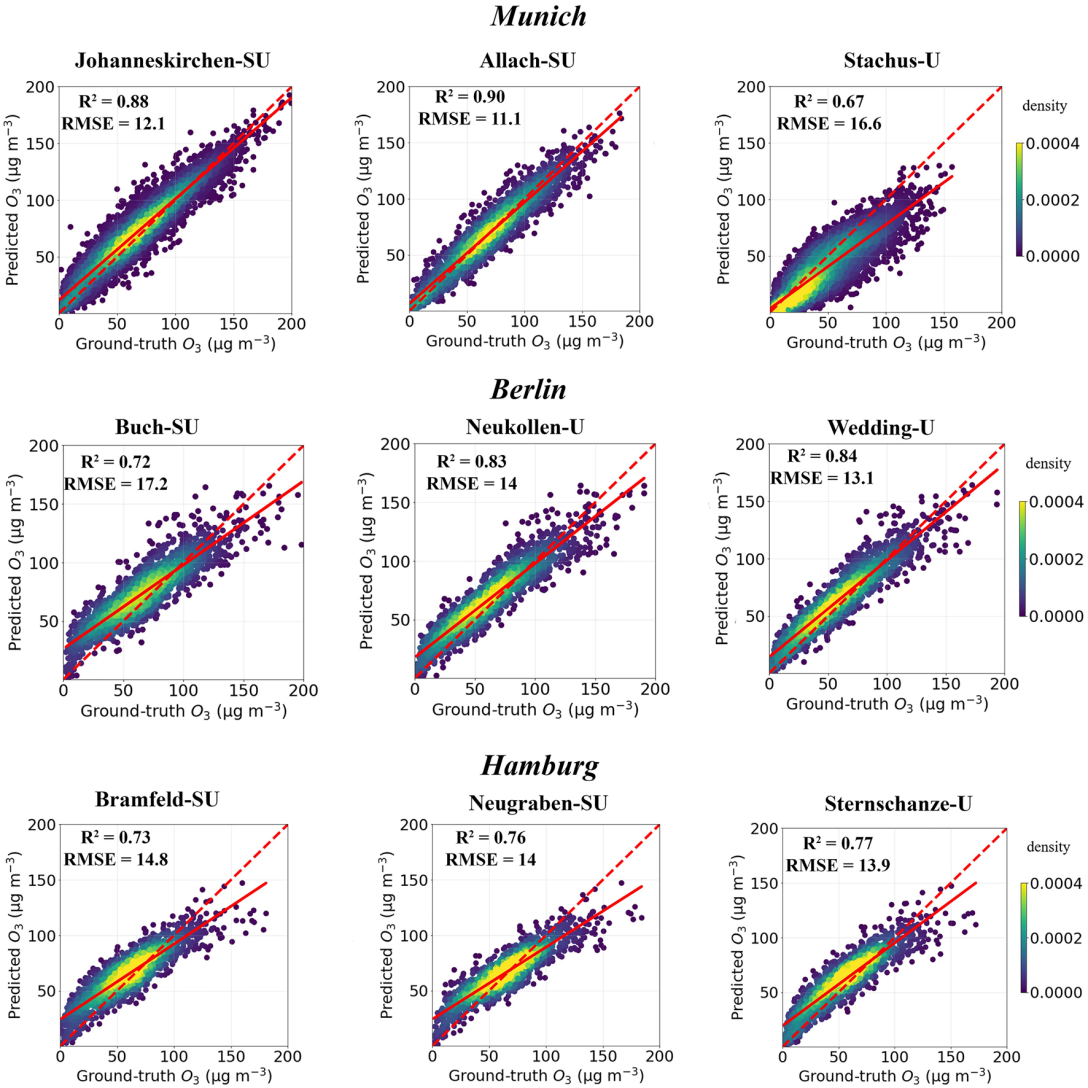
Density scatter plots of predicted ozone by “ML_s_rh_t_wd_blh_no” trained for Lothstrasse station at Munich vs ground-truth ozone measurements for different locations. First row shows the stations for Munich, second row for Berlin and third row for Hamburg stations. U represents urban station and SU represents suburban station. Red solid line represents the linear fit and red dotted line represents 1:1 line.

First, we use the “ML_met_ds” trained for “Lothstrasse” station (5375 days) to predict the ozone concentrations of other three stations in Munich, two (Johanneskirchen, Allach) of which are sub-urban and remaining one (Stachus) is urban station. When compared to ground-truth, the performance of “ML_met_ds” for two sub-urban station is better (R$^{2}$ = 0.86, 0.81 and RMSE = 12.6, 15.1 $\upmu$g m$^{-3}$) than for the urban station (R$^{2}$ = 0.5 and RMSE = 20.3 $\upmu$g m$^{-3}$) (Fig. S6). The predictions are better in all terms when we use “ML_s_rh_t_wd_blh_no”, compared to “ML_met_ds”, indicating that including precursor information plays an important role in explaining ozone variability of other locations (Fig. 3). These findings also imply that ML algorithm trained on long-term data for urban stations are transferable not only to other urban stations, but also to sub-urban stations, which have different emission scenarios, such as low NO$_X$. It could be because a machine learning algorithm trained on long-term data from urban stations can learn ozone variability for various emission scenarios (e.g., low emission activities such as public holidays, weekend, etc.). When including CAMS with “ML_s_rh_t_wd_blh_no” (“ML_s_rh_t_wd_blh_no_cams”), ML model show slightly better performance (Fig. S7).

Similarly, we use the “ML_met_ds”, “ML_s_rh_t_wd_blh_no” and “ML_s_rh_t_wd_blh_no_cams” trained for “Lothstrasse” station to predict the ozone concentration of two major German cities (3 stations for each city) (Figs. 3, S6, S7). Here, as well, the performance of “ML_s_rh_t_wd_blh_no” is better than “ML_met_ds” in all terms, with R$^{2}$ ranges from 0.72 to 0.84 and RMSE ranges from 13.1 to 17.2 $\upmu$g m$^{-3}$. When using “ML_s_rh_t_wd_blh_no_cams”, the performance is slightly better than “ML_s_rh_t_wd_blh_no” in terms of R$^{2}$ and RMSE. We also performed a ML simulation for the days that have OMI data for all nine stations in Munich, Berlin and Hamburg (Tables S3–S5). In all cases, “ML_met_ds_satellite” trained for “Lothstrasse” station performs slightly better than “ML_met_ds” in predicting the ozone concentrations of other locations.

## Discussion

In this study, the potential of a machine learning algorithm in simulating urban surface ozone has been demonstrated. As ozone is primarily produced by complex photo-chemical reactions in the atmosphere, the performance of the ML algorithm with meteorology information alone is promising; however, including the precursor emission (NO$_X$), particularly NO concentration, information further enhance the ML model’s performance in predicting the surface ozone. It could be because NO is an important scavenger of O$_3$ in the urban environment. Due to the scarcity of measurements, we did not use another important insitu ozone precursor (VOC) information in this study, but instead used satellite column HCHO information in the ML model. The addition of a satellite ozone precursor (column NO$_2$, HCHO) information as a new feature has little effect on the ML model performance. This could be because satellite column NO$_2$ and HCHO retrievals are less sensitive to surface emissions. Furthermore, the coarser resolution of satellite retrievals might limit its applicability. This study also reveals that ML algorithm, with O$_3$, meteorology and precursor information (NO), trained for one location can be used to suitably model the surface ozone concentrations of different locations with sparse ozone measurements. However, the performance of ML model vary by location because other factors also influence ozone production. Therefore, we advocate for additional research that focuses on specific campaigns that measure all other factors (such as VOC emissions and aerosol load) influencing ozone formation and use an ML model to simulate the ozone variability of other locations.

## Supplementary Information

Supplementary Information.

## Data Availability

The satellite OMI NO$_2$ and HCHO data can be found at https://disc.gsfc.nasa.gov/. Hourly NO$_2$, NO, CO and O$_3$ concentrations are downloaded from European Environment Agency (EEA) website (https://discomap.eea.europa.eu/map/fme/AirQualityExport.htm). Hourly ERA 5 meteorological data are freely available at https://cds.climate.copernicus.eu/. CAMS global reanalysis surface ozone simulations are obtained from CAMS data store (https://ads.atmosphere.copernicus.eu/).
