# Supplementary material for: Importance of ozone precursors information in modelling urban surface ozone variability using machine learning algorithm
Source: Sci Rep. 2022 Apr 5;12:5646. doi: 10.1038/s41598-022-09619-6 (PMC8983660; doi:10.1038/s41598-022-09619-6)
Supplement: Supplementary file 1 — Supplementary Information. [file 41598_2022_9619_MOESM1_ESM.pdf]

Supplementary material: submission to Scientific Reports

Importance of ozone precursors information in modelling urban surface ozone variability using machine learning algorithm

Vigneshkumar Balamurugan[1,*], Vinothkumar Balamurugan[2], and Jia Chen [1,*]

[1]Environmental Sensing and Modeling, Technical University of Munich (TUM), Munich, 80333, Germany.

[2]Mechanical Engineering, St. Joseph's Institute of Technology, Chennai, 600119, India.

*vigneshkumar.balamurugan@tum.de, jia.chen@tum.de

Table S1. Performance comparisons of different ML simulation types using RF model for Lothstrasse station at Munich. In a total of 5375 days (between 2003 to 2017), first 3800 days used for training and remaining 1575 days used for testing.

ML simulation type	R^2	RMSE	Mean R^2 for K (10) fold CV
**ML_met**	0.79	15.3	0.79
**ML_met_ds**	0.82	14.2	0.82
**ML_insitu**	0.52	23.1	0.54
**ML_met_ds_insitu**	0.88	11.3	0.89
**ML_met_ds_insitu_cams**	0.88	11.4	0.88

Table S2. Sensitivity of predictor variable in "ML_met_ds_insitu" simulation.

Excluded variable	Change in R^2	Change in RMSE	Change in mean R^2 of K (10) fold CV
**Day of week**	0	0	0
**Season**	-0.01	+0.5	-0.01
**Relative humidity**	-0.02	+0.6	-0.02
**Temperature**	-0.04	+1.9	-0.03
**Wind speed**	0	0	0
**Wind direction**	-0.01	+0.4	-0.01
**Boundary layer height**	-0.01	+0.3	0
**In-situ NO_2**	0	-0.1	0
**In-situ NO**	-0.03	+1.4	-0.04
**In-situ CO**	+0.01	-0.2	-0.04

Table S3. Performance comparisons of different ML simulation types (only for days that have OMI data). ML model trained for Lothstrasse station (5375 days) in Munich used to predict the surface ozone concentrations of three measurement stations in Munich.

ML simulation type	Johanneskirchen (sub urban)	Allach (sub urban)	Stachus (urban)
**ML_met_ds**	R^2 = 0.91<br>RMSE = 9.8	R^2 = 0.87<br>RMSE = 12.7	R^2 = 0.43<br>RMSE = 22
**ML_met_ds_satellite**	R^2 = 0.91<br>RMSE = 9.8	R^2 = 0.91<br>RMSE = 10.9	R^2 = 0.44<br>RMSE = 21.9

Table S4. Performance comparisons of different ML simulation types (only for days that have OMI data). ML model trained for Lothstrasse station (5375 days) in Munich used to predict the surface ozone concentrations of three measurement stations in Berlin.

ML simulation type	Buch (sub urban)	Neukollen (urban)	Wedding (urban)
**ML_met_ds**	R^2 = 0.47<br>RMSE = 22.1	R^2 = 0.55<br>RMSE = 21.6	R^2 = 0.65<br>RMSE = 18.5
**ML_met_ds_satellite**	R^2 = 0.47<br>RMSE = 22.3	R^2 = 0.57<br>RMSE = 21.2	R^2 = 0.67<br>RMSE = 18

Table S5. Performance comparisons of different ML simulation types (only for days that have OMI data). ML model trained for Lothstrasse station (5375 days) in Munich used to predict the surface ozone concentrations of three measurement stations in Hamburg.

ML simulation type	Bramfeld (sub urban)	Neugraben (sub urban)	Sternschanze (urban)
**ML_met_ds**	R^2 = 0.11<br>RMSE = 27	R^2 = 0.01<br>RMSE = 28.5<br>Slope = 0.55	R^2 = 0.31<br>RMSE = 23.7<br>Slope = 0.55
**ML_met_ds_satellite**	R^2 = 0.28<br>RMSE = 24.2	R^2 = 0.20<br>RMSE = 25.5	R^2 = 0.47<br>RMSE = 20.6

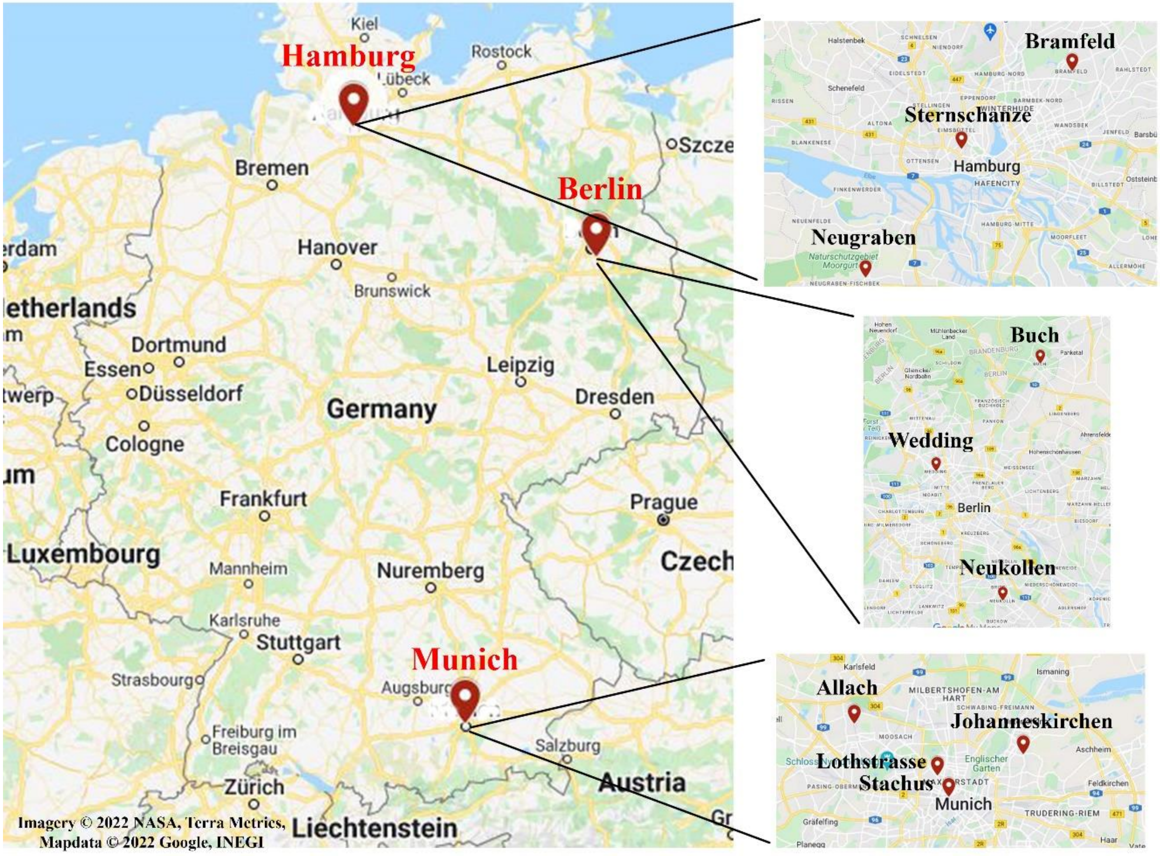

Figure S1. Geographical locations of three German metropolitan areas (Munich, Berlin and Hamburg) and its monitoring stations considered in this study. This map is created using "Google My Maps (https://mymaps.google.com)" tool under the guidelines provided by Google Maps (https://about.google/brand-resource-center/products-and-services/geo-guidelines/#general-guidelines).

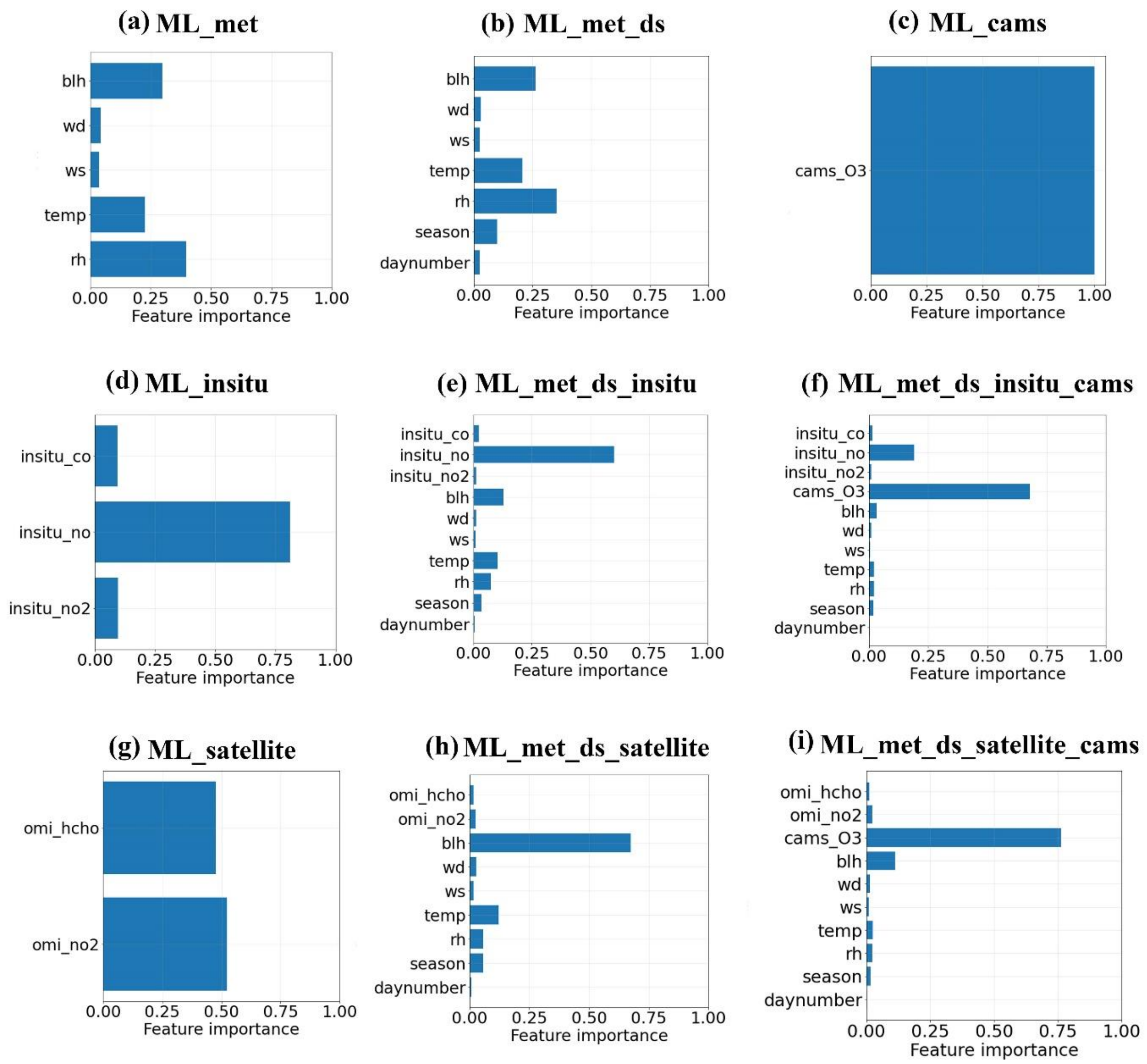

Figure S2. Feature importance derived from sklearn python library's "feature_importance" function for different ML simulation types. (a-f) show the feature importance for the 5375 days case. (g-f) show the feature importance for the 689 days case.

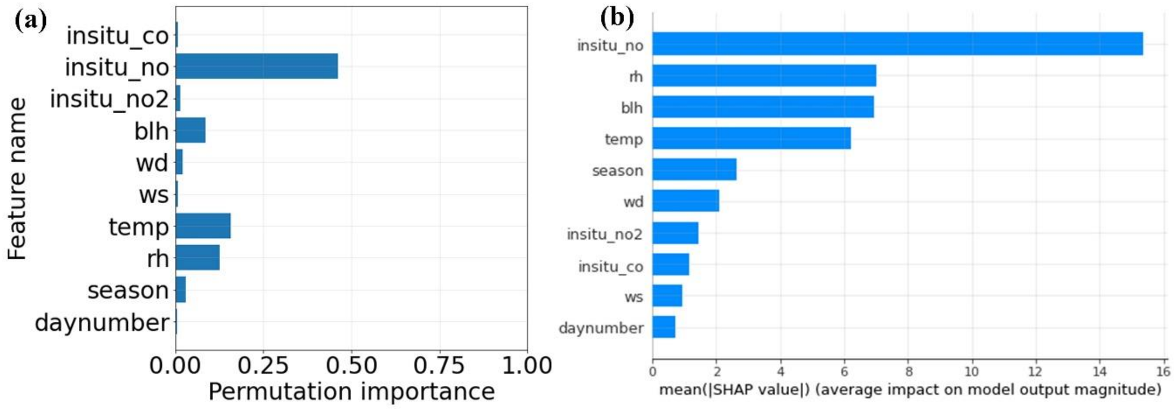

Figure S3. Feature importance derived based on permutation and SHAP values for the case "insitu_met_ds_insitu".

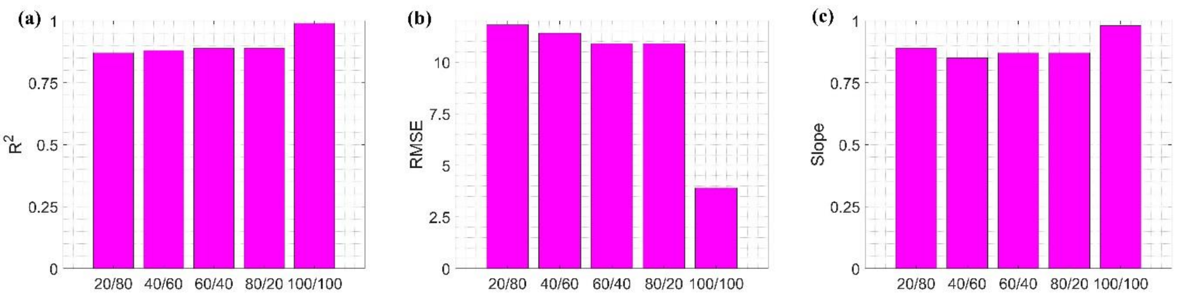

Figure S4. Performance comparisons of different percentage of training/testing dataset for "ML_met_ds_insitu" simulation with 5365 days of measurements at Lothstrasse station in Munich.

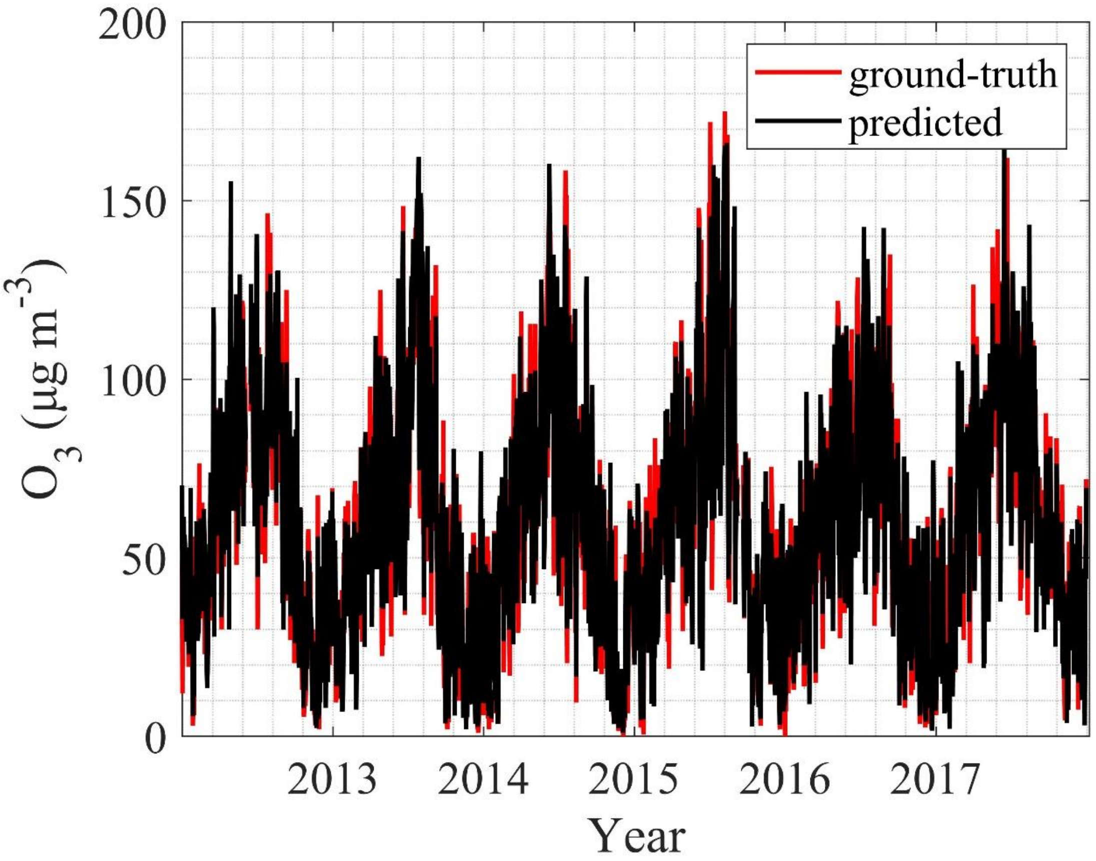

Figure S5. Time-series plot of ground-truth and predicted ozone by “ML_s_rh_t_wd_blh_no” for lothstrasee station.

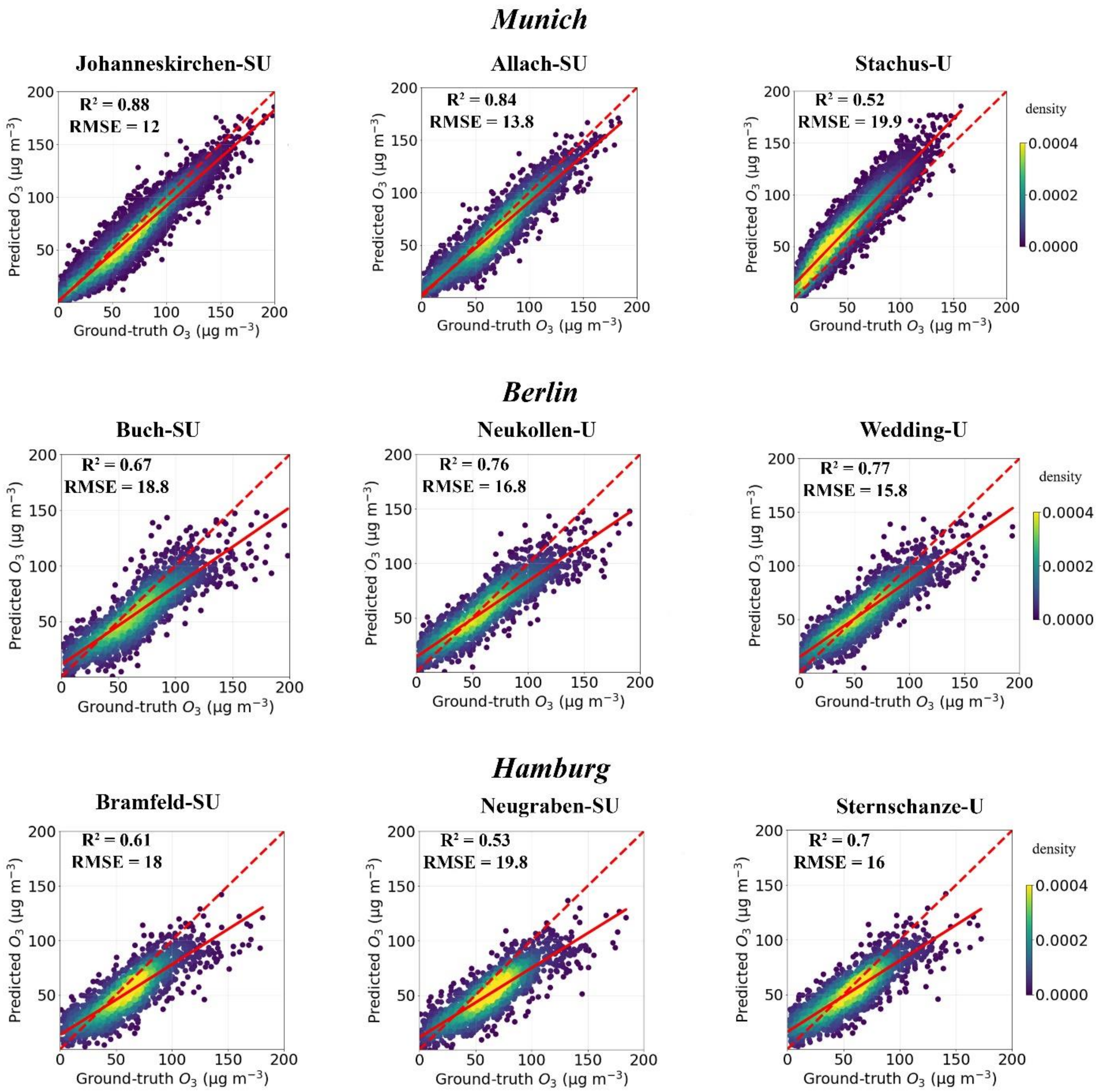

Figure S6. Density scatter plots of predicted ozone by “ML_met_ds” trained for Lothstrasse station at Munich vs ground-truth ozone measurements for different locations. First row shows the stations for Munich, second row for Berlin and third row for Hamburg stations. U represents urban station and SU represents suburban station. Red solid line represents the linear fit and red dotted line represents 1:1 line.

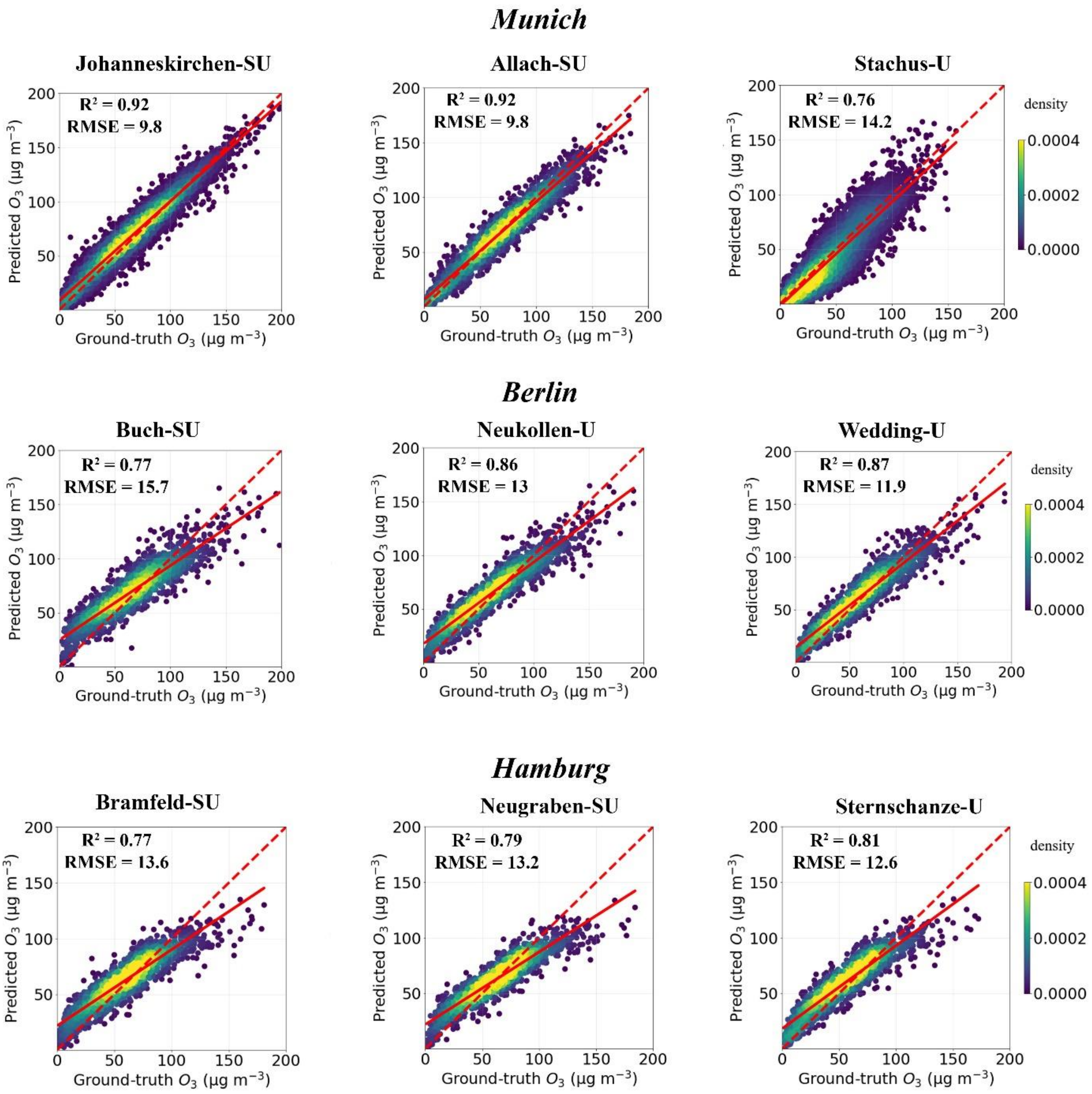

Figure S7. Density scatter plots of predicted ozone by "ML_s_rh_t_wd_blh_no_cams" trained for Lothstrasse station at Munich vs ground-truth ozone measurements for different locations. First row shows the stations for Munich, second row for Berlin and third row for Hamburg stations. U represents urban station and SU represents suburban station. Red solid line represents the linear fit and red dotted line represents 1:1 line.
